# Optimal large‐scale CD34+ enrichment from a leukapheresis collection using the clinimacs prodigy platform

**DOI:** 10.1002/ccr3.3232

**Published:** 2020-08-18

**Authors:** Oscar M. Pello, Diego Lanzarot, Mercedes Colorado, Cristina Amunarriz, Andrés Insunza, Lorena Álvarez‐Rodríguez, María Díez de Velasco, Natividad Sainz‐Sainz, José Luis Arroyo

**Affiliations:** ^1^ Blood and Tissue Bank of Cantabria Marqués de Valdecilla Foundation Santander Spain; ^2^ Haematologic Neoplasms and Haematopoietic Stem Cells Transplantation Group Marqués de Valdecilla Research Institute Santander Spain; ^3^ Miltenyi Biotec Spain Bergisch Gladbach Spain; ^4^ Hematology Department Marqués de Valdecilla Hospital Santander Spain

**Keywords:** CS34+ selection, CliniMACS PRODIGY, hematopoietic stem cell transplant

## Abstract

Optimization of Hematology Patient's treatment: It is possible to obtain a 100% CD34+ recovery after CD34+ selection using the CliniMACS Prodigy.

## Graphical abstract

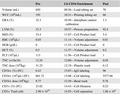

## 1 INTRODUCTION

The CliniMACS Prodigy offers a protocol for CD34+ cell selection from apheresis products. Optimal CD34+ selection with the CliniMACS Prodigy represents a very attractive improvement because is a fully automated device with limited operator involvement. We have performed CD34+ cell enrichment on the CliniMACS Prodigy with 100% CD34+ recovery.

CD34+ enriched hematopoietic progenitors can be used to boost, without prior conditioning, poor graft function after allogeneic hematopoietic stem cell transplantation (allo‐HSCT).^1^ CD34+ cell selection is a standard procedure to deplete T cells from the donor graft to limit graft‐versus‐host disease (GvHD).^1^ Accordingly, current cell selection methods include reducing the number of unwanted T cells (negative selection) and/or enriching for CD34+ cells (positive selection) using anti‐CD34 monoclonal antibodies conjugated with immunomagnetic beads. The CliniMACS Plus semi‐automatic instrument from Miltenyi Biotec has been used for decades for clinical‐scale enrichment of CD34+ cells in a closed and sterile system.^1^ Miltenyi Biotec has recently developed a successor to the CliniMACS Plus, the CliniMACS Prodigy, which is a fully automated device with limited operator involvement. The device automatically performs all the steps of the process, including cell separation, washing, and elution, following standardized protocols. It has been reported that the CliniMACS Prodigy is capable of isolating and even manufacturing various types of advanced therapy medicinal products, complying with the good manufacturing practices laid down by the corresponding regulatory agencies for the production of several cell therapies, including viral‐specific T cells and chimeric antigen receptor T cells.^2^, ^3^ The CliniMACS Prodigy platform contains a preinstalled protocol for immunomagnetic CD34+ cell isolation. We are aware of some centers that have compared the two devices and found that the amount of CD34+ cells obtained with the CliniMACS prodigy was less than with the CliniMACS plus (unpublished results). These centers have elected to continue performing CD34+ selection on the CliniMACS Plus device and this has likely occurred in other centers. Looking into bibliography, three studies from two different groups published their validations comparing the CliniMACS Prodigy and the CliniMACS Plus.^4^, ^5^, ^6^ Although the purity of the CD34+ cells obtained were within accepted clinical limits on both platforms, the quantity of recovered CD34+ cells was similar or lower on the CliniMACS Prodigy while the reduction in T lymphocytes less efficient on this device compared to the CliniMACS Plus.

## 2 CASE REPORT

A 18‐year‐old female patient (40 kg) with MDS‐EB‐2 (myelodysplastic syndrome with excess blasts) with lower cellularity than expected based upon the patient's age underwent allo‐HSCT using cells obtained by leukapheresis from a 100% HLA‐matched unrelated donor (ABO A+/O+; CMV+/−), with a CD34+ cell dose of 1.48 × 10^6^/kg. The patient was conditioned with busulfan (3.2 mg/kg × 4) and cyclophosphamide (120 mg/kg total dose) and the immunosupressors, cyclosporine and methotrexate. On day 90 of the procedure, blood counts were 0.4 × 10^9^/L for neutrophils and <20 × 10^9^/L for platelets (with daily platelets support), with 100% donor chimerism. Because of the evident neutropenia, combined with gastrointestinal GvHD grades III‐IV, we sought to boost the allo‐HSCT with CD34+‐enriched cells. As there was a CliniMACS Prodigy available in our clean room, we used this platform and estimated an initial amount of ~10 × 10^6^/kg CD34+ cells, expecting to obtain 5‐6 × 10^6^/kg of cells needed for the transplant. Two days were required for collection of donor products, which were pooled for analysis in a final volume of 650 mL. Automated blood cell counting gave a total white cell count (WCC) of 193 × 10^6^/mL. Flow cytometry analysis showed 601.72 × 10^3^ CD34+/µL and 0.43% CD34+ cells, giving an initial CD34+ dose of 9.77 × 10^6^ cells/kg and 23.82% CD3+ T lymphocytes (Table 1). The CliniMACS Prodigy system included a TS310 tubing set, 2 vials of CliniMACS CD34 Reagent (monoclonal CD34 antibody coupled to superparamagnetic nanobeads), and 10 mL of human IgG (Flebogamma, 5%). Washing buffer was CliniMACS PBS/EDTA 0.5% human albumin solution (3 L), and cells were eluted in NaCl 0.5% human albumin solution. Installation took 1 hour and CD34+ selection took 5 hours (see Table 1). During the process, the platform alerted us with a “process out of specification” alarm indicating: “Based on entered WBC concentration, target cell frequency, and product volume, the resulting labeled cell count and/or total WBC count exceeded the specification of large scale LP‐34 enrichment process.” We confirmed the warning and started the process out of specification (Table 1). The final product was eluted in 70 mL of elution buffer. We removed a sample for sterility testing and quality controls. Postselection automated cell count gave a total WCC of 6.6 × 10^6^/mL. Flow cytometry analysis showed 5577.66 × 10^3^ CD34+/µL and 96% CD34+ cells, giving a postselection CD34+ dose of 9.76 × 10^6^/kg and consequently 100% CD34+ recovery. Postselection CD3+ was 0.23% (4.45 Log reduction in total CD3 cells) (Table 1). Forty milliliters of this product were infused 2 hours after selection (CD34+ dose of 5.76 × 10^6^/kg and 15 000 CD3+ cells/kg). The remaining 30 mL (CD34+ dose of 4 × 10^6^/kg) was cryopreserved. Fourteen days after CD34+ boost, the patient showed reconstitution with neutrophils at 7.4 × 10^9^/L and platelets at 97 × 10^9^/L, without support.

**TABLE 1:** Product analysis pre‐ and postselection and program steps

	Pre	LS‐CD34 Enrichment	Post
Volume (mL)	650	09:56—Load tubing set	70
WCC (10^6^/mL)	193	10:31—Priming tubing set	66
GRA (%)	32.3	10:49—Interphase camera calibration	1.3
LYM (%)	33.3	10:57—Process preparation	92.4
MID (%)	33.4	11:07—Cell Product load	5.4
RBC (10^6^/µL)	0.03	11:14—Volume adjustment	0.01
HGB (g/dL)	0	11:33—Cell Product load	0
HCT (%)	0.5	11:37—Volume adjustment	0.2
PLT (10^3^/µL)	113	11:56—Cell Product load	1
TNC (×10e10)	12.54	12:00—Volume adjustment	0.05
TNC dose (10^8^/kg)	31.25	12:19—Platelet wash	0.12
CD34+ (%) (FC)	0.43	13:07—IgG labeling	96
CD34+ (10^3^/µL) (FC)	601.72	13:08—Cell labeling	5577.66
CD34+ dose (10^6^/kg)	9.77	13:39—Bead wash	9.76
CD3+ (%) (FC)	23.82	14:43—Cell filtration	0.23
CD3+ Total cells	2.98 × 10^10^	14:55—Cell separation	1.06 × 10^6^

Note

WCC (white cell count), GRA (granulocytes), LYM (lymphocytes), MID (middle‐size cells), RBC (Red blood cells), HGB (Hemoglobin), HCT (Hematocrite), PLT (platelets) were obtained with an automated cell counter Cell‐Dyn Emerald 22. Flow cytometry (FC) analysis for CD34+%, CD34+ counts, and CD3+% was performed in a Beckman‐Coulter Navios. Total Nucleated Cells (TNC) and CD34+ doses were calculated for 40 kg patient's weight.

We sought to identify the parameters that may play a role in the positive outcome of the CD34+ cell selection process to benefit patients in need of this cell product. The CD34+ enrichment program in the CliniMACS Prodigy software allows two different scales: normal (0.6 × 10^9^ target cells/ 6 × 10^10^ total cells) and large (1.2 × 10^9^ target cells/ 12 × 10^10^ total cells), both in a volume 50‐660 mL. Compared with the previous validations of CD34+ selections using the CliniMACS Prodigy, our attention was drawn to the large initial volume of cells. As is common when validating a new technique or methodology, the optimal starting material collections were used (6 × 10^10^ total cells for normal scale or 12 × 10^10^ total cells for large scale, with a relatively high percentage of CD34+ cells in the smallest possible volume). Platelets in the starting product are believed to decrease cell recovery by interfering with the binding between the immunomagnetic reagent and the CD34+ cells,^7^ and for this reason the program includes a platelet removal step. When starting with a small volume, the entire product is loaded and only one volume adjustment is performed before the program moves to the platelet wash step. In this context, our starting material could be considered nonideal, as the CliniMACS Prodigy needed to load the product three times (Table 1). Each time, the volume was adjusted, cells were concentrated and supernatants removed. Accordingly, more platelets were removed in each step. It is possible that superior results are obtainable with more diluted leukapheresis collections, in which more product loading and volume adjustments are needed. We would encourage centers using the CliniMACS Prodigy to evaluate larger and less concentrated starting materials to test the reduction in platelets and, consequently, its positive impact on selection efficiency.

## CONFLICT OF INTEREST

DL is an employee of Miltenyi Biotech. The rest of the authors declare no conflict of interest.

## AUTHOR CONTRIBUTIONS

OMP, DL, LAR, MDV, and NSS: involved in cell selection. AI: performed flow cytometry analysis. MC, CA, and JLA: supervised cell apheresis, cell infusion, and patient evolution. OMP: wrote the manuscript. All authors: reviewed the manuscript.

## ETHICAL APPROVAL

All procedures performed in studies involving human participants are in accordance with the ethical standards of the institutional and/or national research committee and with the 1964 Helsinki declaration and its later amendments or comparable ethical standards.
